# Case Report: Glucocorticoids with Early Aggressive Rituximab and Upadacitinib regimen in the treatment of three patients with anti-MDA5 dermatomyositis-associated interstitial lung disease

**DOI:** 10.3389/fmed.2026.1896522

**Published:** 2026-07-28

**Authors:** Edmund Ka Ming Wong, Lydia Ho Pui Tam, Jacqueline So, Tommy Tsz On Lam, Man Yui Choi, Lai-Shan Tam, Ho So

**Affiliations:** 1 Department of Medicine and Therapeutics, Prince of Wales Hospital, Hong Kong, Hong Kong SAR, China; 2 Department of Medicine and Therapeutics, Faculty of Medicine, The Chinese University of Hong Kong, Hong Kong, Hong Kong SAR, China

**Keywords:** anti-MDA5, combination therapy, dermatomyositis, interstitial lung disease, rituximab, upadacitinib

## Abstract

**Background:**

Anti-melanoma differentiation-associated gene 5 (anti-MDA5) dermatomyositis (DM) is a severe phenotype that is frequently complicated by rapidly progressive interstitial lung disease (RP-ILD), which carries high mortality despite conventional immunosuppression. Given the dual pathogenesis involving B-cell activation and dysregulated interferon (IFN) pathways, there is a rationale for combining rituximab with Janus kinase (JAK) inhibitors.

**Methods:**

We present three patients with anti-MDA5 DM and severe ILD who were treated at a single center with a novel aggressive triple regimen consisting of high-dose glucocorticoids, rituximab, and upadacitinib. Patients were reviewed for clinical, biochemical, and radiological responses.

**Results:**

All three patients demonstrated significant clinical and objective improvement following the initiation of the glucocorticoid-rituximab-upadacitinib regimen, including resolution of skin disease, improvement in lung function and high-resolution computed tomography (HRCT) findings, and reduction in disease activity markers. No deaths were observed during at least 6 month of follow-up. Serious opportunistic infections occurred in one patient. Concomitant intravenous immunoglobulin (IVIG) therapy was administered to one patient with dysphagia and to one patient with superimposed infection.

**Conclusion:**

An early aggressive regimen of dual-pathway inhibition combining rituximab and upadacitinib, in addition to high-dose glucocorticoids and adjunctive IVIG therapy, may be an effective therapeutic strategy for severe anti-MDA5-associated ILD.

## Introduction

Anti-melanoma differentiation-associated gene 5 (anti-MDA5) dermatomyositis (DM) represents a distinct and aggressive phenotype within idiopathic inflammatory myopathies. First described in 2005, it is characterized by the presence of autoantibodies targeting MDA5, a cytoplasmic RNA helicase involved in innate antiviral immunity (1). Clinically, patients often present with characteristic cutaneous manifestations, including Gottron’s papules, heliotrope rash, cutaneous ulcerations and palmar papules but with little to no clinically significant muscle weakness, defining the condition as a form of clinically amyopathic dermatomyositis (CADM) (2, 3). The gravest complication of anti-MDA5 DM is the development of rapidly progressive interstitial lung disease (RP-ILD), which is the primary determinant of mortality. The prognosis is particularly poor, with a reported six-month mortality rate of 43% from disease onset (4).

The pathogenesis of anti-MDA5 DM is believed to involve a dysregulated type I interferon (IFN) pathway, mediated by MDA5 activation leading to pro-inflammatory cytokine production (5). This IFN signature correlates with disease activity and poor prognosis in ILD (6, 7). In addition, B-cell activation and autoantibody production, which are not confined to anti-MDA5 antibodies, are also central to the disease process and trigger direct IFN-gamma (type II IFN) secretion by peripheral blood cells (8).

The recommended treatment regimen for anti-MDA5 DM with RP-ILD aims to halt the rapid immunological assault on the lungs. First-line therapy involves high-dose glucocorticoid (e.g., methylprednisolone 500 mg daily for 3 to 5 days) to induce rapid immunosuppression. This is combined with a calcineurin inhibitor, with or without intravenous cyclophosphamide (9). Despite intensive multi-agent immunosuppression, a significant proportion of patients develop refractory disease, highlighting a critical unmet need for more effective therapeutic strategies (5).

Given the dual pathogenic pathways of B-cell activation and IFN-driven inflammation, there is a strong rationale for combining targeted therapies. Rituximab, a monoclonal antibody that depletes CD20 + B cells, has shown promise in refractory cases of anti-MDA5 DM, with reports of improved survival and lung function (10, 11). Janus kinase (JAK) inhibitors also represent a promising strategy by blocking the downstream signaling of IFN and other pro-inflammatory cytokines implicated in the disease (12). Prospective cohorts, case reports and case series suggest the potential efficacy of JAK inhibition in stabilizing anti-MDA5 related RP-ILD (13–15). This case series describes our experience and outcomes in treating severe, rapidly progressive anti-MDA5-associated ILD with a novel combination regimen of rituximab and a JAK1 selective inhibitor, added to conventional immunosuppressive therapy. We aim to contribute to the evolving evidence on targeted dual-pathway inhibition as a salvage and potential therapeutic option for patients predicted to have poor prognosis or rapidly progressive disease.

## Case description

### Case 1

A 42-year-old non-smoking Chinese woman with no major medical history presented with a four-week history of progressive dyspnea on exertion, non-productive cough and a 4.5 kg weight loss over 1 month. Physical examination showed heliotrope rash, Gottron’s papules, and ulcerative rash over the elbows, while muscle power was normal. Serological evaluation revealed strongly positive anti-MDA5 and anti-Ro52 antibodies, while antinuclear antibody and anti-extractable nuclear antigen testing were negative. Positron emission tomography–computed tomography (PET-CT) demonstrated multiple areas of pulmonary fibrosis. Based on these findings, an initial diagnosis of anti-MDA5-positive CADM with associated ILD was made. Treatment was initiated with oral prednisolone 40 mg daily (1 mg/kg/day), two 1-gram doses of rituximab administered 2 weeks apart, and oral tacrolimus 2 mg twice daily. The prednisolone was tapered by 5 mg every 2 weeks. Despite this triple immunosuppressive therapy, the patient’s symptoms worsened over the following 6 weeks, with increased dyspnea and persistent skin involvement, necessitating hospital admission, while she was on prednisolone 30 mg daily.

On admission, she required supplemental oxygen at 2 liters per minute. Physical examination revealed Gottron’s papules, inverse Gottron’s sign, and periungual erythema. Again, no muscle weakness was detected. High-resolution computed tomography (HRCT) of the thorax showed diffuse peribronchial consolidative changes and underlying fibrotic changes with traction bronchiectasis, consistent with a mixed pattern of organizing pneumonia and nonspecific interstitial pneumonia (NSIP) (Figure 1). Bronchoscopy with bronchoalveolar lavage (BAL) was non-diagnostic of any infection. Spirometry demonstrated a forced vital capacity (FVC) of 50% of the predicted value. Laboratory studies revealed an elevated serum ferritin level of 710 pmol/L (reference: 29–337 pmol/L), lactate dehydrogenase (LDH) of 347 U/L (reference: 103–199 U/L), and lymphopenia (0.3 × 10^9^/L; reference: 1.1–3.1 × 10^9^/L), while C-reactive protein (CRP) and creatine kinase (CK) level were normal. A diagnosis of anti-MDA5-positive DM with RP-ILD was confirmed.

**Figure 1:**
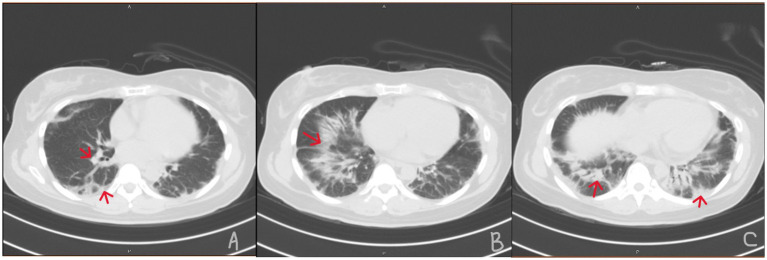
HRCT thorax showing diffuse peribronchial consolidative changes (red arrow) in the upper-to-lower lung zones **(A–C)**.

The treatment regimen was escalated. The patient received a three-day course of intravenous methylprednisolone 500 mg daily, followed by oral prednisolone 50 mg daily on a tapering schedule. Tacrolimus was discontinued, and upadacitinib 15 mg once daily was initiated. Following the introduction of upadacitinib, the patient’s rash and dyspnea improved markedly. Serum ferritin and LDH levels normalized within 4 weeks. An HRCT thorax performed 8 weeks later showed resolution of the consolidative changes, with only minimal residual ground-glass opacities (GGO). Lung function improved significantly, with FVC increasing to 76% of the predicted value at the eight-week follow-up. The patient successfully tapered prednisolone to 7.5 mg daily and was weaned off supplemental oxygen therapy 4 months after starting upadacitinib.

### Case 2

A 59-year-old Chinese man, a taxi driver and known hepatitis B virus carrier on entecavir, presented with shortness of breath on exertion, a non-healing tongue ulcer and a progressive cutaneous eruption over the face, elbows and hands for 1 month. His past medical history was otherwise unremarkable. Physical examination revealed extensive cutaneous involvement strongly suggestive of DM, including a heliotrope rash, violaceous plaques, Gottron’s papules, inverse Gottron’s sign and ulcerative rash over the elbows. He had marked hoarseness but had no objective muscle weakness. Speech therapist assessment identified dysphagia with an aspiration risk. Serological testing revealed strongly positive anti-MDA5 and anti-Ro52 antibodies. HRCT of the thorax demonstrated patchy subpleural reticulation, atelectasis, and ground-glass infiltrates in the bilateral mid-to-lower lung zones, consistent with an NSIP pattern (Figure 2). Pulmonary function tests showed a restrictive defect, with FVC reduced to 55% of the predicted value and an adjusted diffusing capacity for carbon monoxide (DLCO) of 41% predicted. Laboratory studies revealed a markedly elevated serum ferritin level of 7,417 pmol/L (reference: 29–337 pmol/L), an elevated LDH of 343 U/L (reference: 103–199 U/L), and lymphopenia (0.3 × 10^9^/L; reference: 1.1–3.1 × 10^9^/L). CRP and CK levels were within normal limits. A diagnosis of anti-MDA5 CADM with ILD was established.

**Figure 2:**
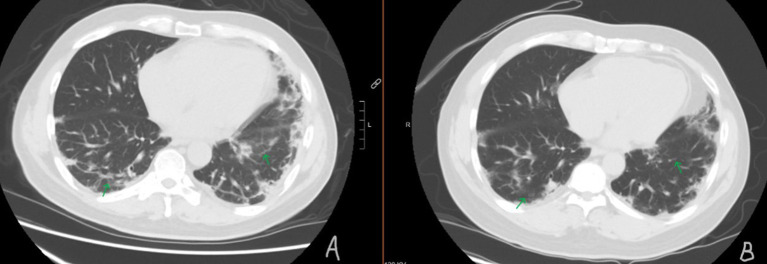
HRCT showing ground-glass infiltrations in the bilateral mid-to-lower lung zones (**A**, green arrow), and interval improvement 6 months after treatment (**B**, green arrow).

Treatment was initiated with oral prednisolone 70 mg daily (1 mg/kg/day) on a tapering schedule, two 1-gram doses of rituximab administered 2 weeks apart, and upadacitinib 15 mg daily. Given his dysphagia and aspiration risk, six monthly courses of intravenous immunoglobulin (IVIG) at 2 g/kg over 2 days were added as adjunctive therapy.

At the six-month follow-up, the patient had complete resolution of the rash and dysphagia, and speech therapist evaluation deemed him fit to resume a usual diet. He had successfully tapered prednisolone to 5 mg daily. Follow-up HRCT of the thorax showed interval reduction in GGO, increased lower-lobe lung volume, and less confluent subpleural fibrosis (Figure 2). Pulmonary function improved, with FVC increasing to 64% predicted and adjusted DLCO to 43% predicted. Serum ferritin decreased significantly to 1,545 pmol/L, and LDH improved to 303 U/L. Lymphopenia persisted at 0.4 × 10^9^/L.

### Case 3

A 61-year-old non-smoking Chinese woman with glucose-6-phosphate dehydrogenase (G6PD) deficiency presented with a five-week history of dyspnea, cough, fever, polyarthralgia, hoarseness, and cutaneous eruption. Physical examination revealed heliotrope rash, periungual erythema, Gottron’s sign over the elbows, inverse Gottron’s papules over the palms, and holster sign on both thighs, without clinical muscle weakness (Figure 3). Laboratory investigations showed an elevated CK of 996 U/L (reference: 26–192 U/L), a markedly elevated serum ferritin of 3,159 pmol/L (reference: 29–337 pmol/L), elevated LDH of 405 U/L (reference: 103–199 U/L) and lymphopenia (0.8 × 10^9^/L; reference: 1.1–3.1 × 10^9^/L). The interferon-gamma release assay (IGRA) was negative. Serology was strongly positive for anti-MDA5 and anti-Ro52 antibodies. Spirometry demonstrated an FVC of 69% predicted value. A PET-CT scan revealed patchy areas of peripheral consolidation in both lungs. A diagnosis of anti-MDA5 DM with ILD was confirmed.

**Figure 3:**
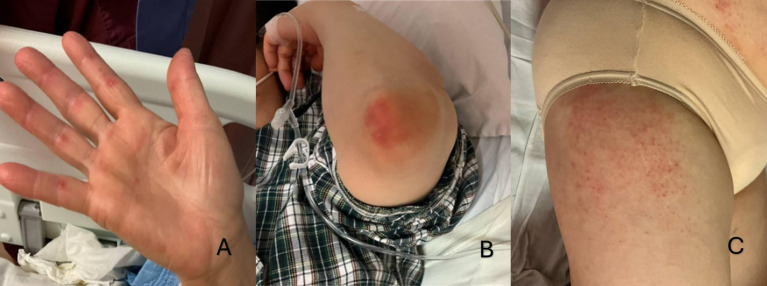
Inverse Gottron’s sign **(A)**, Gottron’s sign over the elbow **(B)**, and holster sign over the thigh **(C)**.

Treatment was initiated with oral prednisolone 60 mg daily (1 mg/kg/day), two 1-gram doses of rituximab administered 2 weeks apart, and tofacitinib 5 mg twice daily. Because of her G6PD deficiency, *Pneumocystis jirovecii* pneumonia (PCP) prophylaxis was initiated with monthly inhaled pentamidine.

Four weeks after starting treatment, she was readmitted with fever, persistent cutaneous eruption, dyspnea, and productive cough, requiring 2 L/min of supplemental oxygen. Despite empirical broad-spectrum antibiotics and IVIG, her respiratory status deteriorated, necessitating transfer to the intensive care unit for high-flow oxygen therapy at 60 L/min with a fraction of inspired oxygen (FiO2) of 60%. HRCT of the thorax showed worsening of the NSIP pattern, with diffuse ground-glass and consolidative changes mixed with coarse reticular opacities in both lungs (Figure 4). Ferritin level increased to 7,984 pmol/L, LDH level increased to 466 U/L, and lymphocyte count decreased to 0.1 × 10^9^/L. Bronchoscopy with BAL was positive for *Pneumocystis jirovecii* on polymerase chain reaction (PCR), and serum 1,3-beta-D-glucan assay was positive. Tofacitinib was withheld. She was treated with a 21-day course of intravenous pentamidine, transitioning to oral atovaquone after CRP normalised. In view of persistent DM rash, high level of ferritin (7,262 pmol/L), and refractory lung disease, upadacitinib 15 mg daily was subsequently initiated. Her condition stabilized after 5 weeks, and she was discharged on long-term oxygen therapy (LTOT) at 2 L/min.

**Figure 4:**
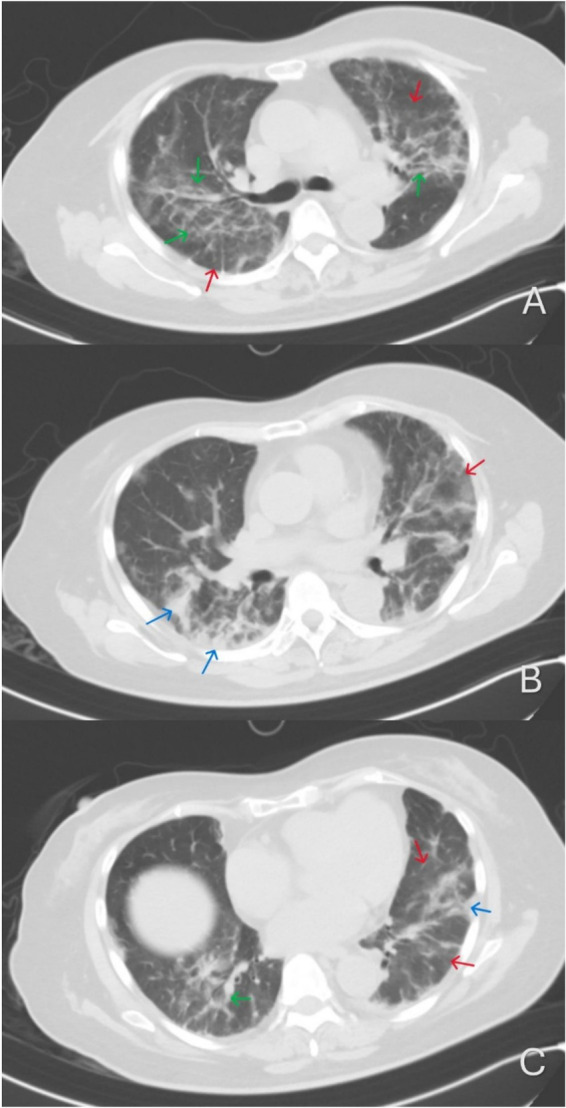
HRCT showing diffuse ground-glass changes (red arrows) and consolidative changes (blue arrows) mixed with coarse reticular opacities (green arrows) in the upper **(A)**, middle **(B)**, and lower **(C)** zones of both lungs.

Eight weeks later, her cutaneous rash had subsided, and her ILD had improved with upadacitinib and tapering prednisolone, at a dose of 20 mg daily. Ferritin improved to 2,168 pmol/L, and LDH was 430 U/L. She was able to reduce her LTOT requirement to 1 L/min.

Four months after her initial presentation, she was admitted again with fever and left knee and right wrist arthritis. Aspirates from both affected joints and sputum samples were positive for acid-fast bacilli (AFB) and *Mycobacterium tuberculosis* PCR, confirming disseminated tuberculosis (TB). Upadacitinib was discontinued, and the patient was continued with oral prednisolone 20 mg daily. Due to rifampin-induced acceleration of corticosteroid metabolism, this 20 mg dose provided a clinical exposure equivalent to approximately 10 mg daily. Anti-tuberculous therapy with isoniazid, rifampin, pyrazinamide, and ethambutol was concurrently initiated.

## Discussion

### Immunopathogenic rationale of therapeutic strategy

MDA5 is an intracellular pattern recognition receptor belonging to the retinoic acid-inducible gene I (RIG-I) family. It typically recognizes viral double-stranded RNA to activate type I IFN signaling (16). In anti-MDA5 DM, profound dysregulation of this system occurs. Autoreactive B cells produce anti-MDA5 autoantibodies that, *in vitro*, directly induce neutrophil extracellular trap (NET) formation (17). These NETs and their components act as potent profibrotic stimuli, promoting lung fibroblast proliferation and subsequent rapid-onset alveolar injury (18). Furthermore, immune complexes formed by anti-MDA5 antibodies and MDA5 autoantigens continuously activate plasmacytoid dendritic cells, driving a massive, self-amplifying cascade of type I IFN production (19). This hyper-interferonemia induces CX3CL1 production by pulmonary vascular endothelial cells, accelerating the recruitment of pathogenic CX3CR1-positive inflammatory cells into the lung parenchyma (20). Concurrently, autoreactive B cells stimulate monocytes to produce IFN-gamma (type II IFN) (8). Recent transcriptomic and proteomic studies have highlighted that an intensified type II IFN signature, alongside elevated levels of downstream interleukins (IL-4, IL-15, and IL-21), closely correlates with disease severity and RP-ILD (21, 22). This multi-lineage pathogenesis underscores why traditional monotherapy or sequential treatments frequently fail. Effectively halting this aggressive disease requires a dual-timeline strategy: immediate, broad-spectrum cytokine blockade to control the acute hyper-inflammatory storm driven by interferons, combined with targeted B-cell depletion to stop the upstream generation of pathogenic autoantibodies and immune complexes.

### Pharmacological selection: why upadacitinib?

Upstream biological agents targeting the type I IFN receptor, such as the anti-IFNAR1 monoclonal antibody anifrolumab, have also gained traction in the treatment of anti-MDA5 disease (23). However, upstream blockade that specifically isolates the type I IFN pathway may have inherent therapeutic limitations in acute, severe anti-MDA5 DM. As noted, anti-MDA5 DM is driven by a complex cytokine network, including type II IFN, IL-4, IL-15, and IL-21, which bypass IFNAR1 entirely (21, 22, 24) but may still be intercepted by JAK inhibition (22, 24).

Although a pioneering cohort from Shanghai using the pan-JAK inhibitor tofacitinib reported 100% six-month survival rate (18 out of 18 patients) in early-stage anti-MDA5-positive ILD (25), a recent large multicenter cohort study reported that combining tofacitinib with glucocorticoids, with or without other immunosuppressants, still yielded a considerable 32.4% (94 of 290) one-year mortality or transplantation rate in anti-MDA5 DM-ILD (13). Limited data from a restrospective cohort and case series also suggest that baricitinib may be efficacious in treating anti-MDA5 DM-ILD patients (26, 27).

The choice of upadacitinib—a second-generation, highly selective JAK1 inhibitor—over first-generation pan-JAK inhibitors such as tofacitinib or baricitinib is driven by its distinct pharmacodynamic profiles. Tofacitinib predominantly blocks JAK1 and JAK3, while baricitinib targets JAK1 and JAK2. Crucially, ex vivo pharmacodynamic assays demonstrate that upadacitinib has three-fold higher selectivity for inhibiting IFN-alpha-related STAT3 signaling over erythropoietin (EPO)-related STAT5 signaling compared to tofacitinib (28). This heightened selectivity maximizes suppression of pathogenic type I and type II IFN pathways, which rely on JAK1/TYK2 and JAK1/JAK2 heterodimers, respectively, while minimizing the off-target myelosuppressive and erythroid toxicities associated with profound, non-selective JAK2 or JAK3 inhibition. A multicenter cohort involving 434 patients with anti-MDA5 DM-ILD demonstrated a trend toward higher lung transplant-free survival in patients treated with upadacitinib compared with tofacitinib (29), although overall data on upadacitinib remain very limited.

### Synergy with rituximab

Although rituximab has been used in refractory DM, high-quality evidence regarding its efficacy in anti-MDA5 DM is lacking. Our current approach builds on our previously reported successful experience using rituximab monotherapy or sequential therapy to treat four patients with anti-MDA5 positive DM and refractory RP-ILD (11). Rituximab achieves structural depletion of CD20 + B cells, effectively reducing the production of anti-MDA5 antibodies and subsequent immune-complex-mediated dendritic cell activation. However, this process takes several weeks to translate fully into clinical remission (30).

As demonstrated by previous prospective trials on combination therapy, a “step-up” strategy—waiting for initial therapies to fail before escalating to a more potent immunosuppressive regimen—carries a high risk of death or irreversible pulmonary fibrosis in rapidly deteriorating patients (31). The clinical success observed in this series stems from the kinetic synergy of the combination: upadacitinib provides an immediate, rapid-acting intracellular brake on the acute cytokine cascade, while rituximab provides definitive, long-term upstream eradication of autoantibody-producing B-cell clones. IVIG was successfully reserved as a pleiotropic adjunct to halt overwhelming immune hyperactivity in patients complicated by dysphagia or active infection (32).

### Therapeutic landscape and clinical outcomes of the case series

The optimal treatment regimen for anti-MDA5 associated ILD remains an ongoing challenge in rheumatology. The regimen used in East Asia—combining high-dose corticosteroids, intravenous cyclophosphamide pulses, and calcineurin inhibitors—has yielded limited success, with a reported six-month mortality rate of 32.1% (67 deaths in 209 patients) in a landmark Japanese cohort (33). Studies of tofacitinib as part of combination treatment with glucocorticoids showed slightly better mortality rates, ranging from 24.1 to 28.3% (13, 27). This suggests that some patient groups, particularly those with poor prognostic factors for RP-ILD and mortality, may benefit from more potent up-front combination treatment with JAK inhibitors.

The clinical course and characteristics of the three patients are summarised in Table 1. All three patients presented with hallmark cutaneous lesions of Gottron’s sign. All developed significant ILD, evidenced by restrictive lung defects on pulmonary function testing and characteristic HRCT findings, including NSIP, GGO, and consolidation. Notably, while all patients demonstrated serological positivity for anti-MDA5, testing was limited to a qualitative line blot immunoassay due to the unavailability of quantitative assays at our center. Despite this testing limitation, a severe systemic inflammatory burden was clearly indicated by elevated serum ferritin and LDH, profound lymphopenia, and anti-Ro52 co-positivity—a combination of factors strongly predictive of rapid deterioration and high mortality (34, 35). Upadacitinib was deployed as a pivotal component of rescue therapy in Cases 1 and 3 after conventional regimens proved insufficient or toxic, and as an upfront, first-line intensive agent in Case 2 to preempt anticipated clinical deterioration. All three patients demonstrated objective signs of improvement, including complete resolution of skin manifestations, rapid reduction in serum disease activity markers (ferritin and LDH), and stabilization or improvement in pulmonary function (FVC) and HRCT thorax features, permitting rapid and safe tapering of systemic corticosteroids.

**Table 1:** Summary of patient characteristics at baseline and clinical course.

Feature	Case 1	Case 2	Case 3
Demographics	42 years old woman	59 years old man	61 years old woman
Observation period	6 months	8 months	7 months
Clinical involvement at baseline			
Skin	+	+	+
Muscle	−	−	−
Lung	+	+	+
Laboratory data (at presentation/ initiation of upadacitinib/ last observation)			
Ferritin (reference: 29–337 pmol/L)	710/593/ 36	7,417/7,417/1,545	3,159/7,984/1,249
LDH (reference: 103–199 U/L)	347/347/ 220	343/343 / 303	405/466/ 203
Lymphocyte count (reference: 1.1–3.1 × 10^9^/L)	0.3/0.3/0.8	0.3/0.3/0.4	0.8/0.1/0.3
Serology	anti-MDA5+, anti-Ro52+	anti-MDA5+, anti-Ro52+	anti-MDA5+, anti-Ro52+
ILD pattern (HRCT)	Mixed organizing pneumonia /NSIP with GGO and consolidation	NSIP with GGO and consolidation	NSIP with GGO and consolidation
Pulmonary function (at presentation/last observation)			
FVC (% predicted)	50% / 76%	55% / 64%	69% / not available
DLCO (% predicted)	Not available / not available	41% / 43%	Not available / not available
Oxygen requirement (at presentation/ initiation of upadacitinib/ last observation)	Room air / nasal cannula 2 L per minute / Room air	Room air / room air / room air	Room air / high-flow therapy with FiO2 0.6 / nasal cannula 1 L per minute
Initial Regimen (before Upadacitinib)	Prednisolone + rituximab + tacrolimus	Prednisolone + rituximab	Prednisolone + rituximab + tofacitinib
Time from symptom onset to upadacitinib initiation	10 weeks	4 weeks	12 weeks
Reason for upadacitinib	Worsening of dyspnea, rash, radiographic progression of ILD	Anticipated very high risk of RP-ILD development	Persistent high disease activity
Adjunctive therapy	None	IVIG	IVIG
Infections	None documented	None documented	PCP pneumonitis; disseminated TB
Clinical outcome	Improved rash, dyspnea, oxygen requirement, steroid requirement, CT findings, and lung function	Improved rash, dysphagia, steroid requirement, CT findings, and lung function	Improved rash, dyspnea, oxygen requirement, and steroid requirement; complicated by pulmonary and articular TB

### Poly-immunosuppressive risks

High rates of infection, including bacterial, viral, and fungal infections, have been reported in patients receiving triple immunosuppression therapy (31). Patients with anti-MDA5 DM have an intrinsic, disease-specific susceptibility to infection, driven by severe native lymphopenia and a profoundly dysregulated immune microenvironment (36). A meta-analysis evaluating the safety of tofacitinib in the treatment of anti-MDA5 DM-ILD reported infection rates ranging from 17 to 22% (37). This series sharply highlights these risks. Despite negative baseline screening and antimicrobial prophylaxis, Case 3 developed concurrent disseminated TB and severe PCP infection. Therefore, while a multi-targeted approach combining upadacitinib and rituximab is highly effective at halting rapid pulmonary progression, it demands extreme clinical vigilance. In our cohort, infection-mitigation strategies included mandatory PCP (oral co-trimoxazole 480 mg daily or inhaled pentamidine 300 mg monthly) and cytomegalovirus (oral valgancyclovir 900 mg daily) prophylaxis for all three patients, which was maintained uninterrupted until the prednisolone dose was successfully tapered to 5 mg per day or less. This prolonged strategy is more stringent than standard EULAR guidelines, which recommend PCP prophylaxis when a prednisolone dose greater than 15 mg/day is taken for more than 2 weeks (38).

To mitigate the profound infectious risks of this intensive regimen, careful patient selection is mandatory. Based on our experience and the molecular rationale, we propose that the GEARUP regimen be reserved as an upfront or early consolidation therapy specifically for anti-MDA5 DM-ILD patients carrying a critical burden of poor prognostic factors. Ideal candidates include those with RP-ILD and an active oxygen requirement, or patients whose initial laboratory panels reveal an overwhelming systemic inflammatory burden marked by elevated serum ferritin, elevated LDH, profound lymphopenia, and anti-Ro52 co-positivity. In this specific subpopulation, waiting for standard therapies to fail causes a substantial risk of mortality, making immediate, aggressive dual-pathway inhibition a viable treatment perspective.

## Conclusion

This case series provides preliminary evidence that the novel dual-pathway inhibition combination of rituximab and upadacitinib, together with high-dose corticosteroids and adjunctive IVIg, may represent a potential therapeutic strategy for severe, progressive anti-MDA5 DM-associated ILD. However, prospective data and controlled trials are needed to confirm the efficacy and safety of the GEARUP (Glucocorticoids with Early Aggressive Rituximab and UPadacitinib) regimen.

## Data Availability

The original contributions presented in the study are included in the article/supplementary material, further inquiries can be directed to the corresponding author.
